# Rare Cytogenetic Abnormalities in Myelodysplastic Syndromes

**DOI:** 10.4084/MJHID.2015.034

**Published:** 2015-05-01

**Authors:** Ulrike Bacher, Julie Schanz, Friederike Braulke, Detlef Haase

**Affiliations:** Department of Hematology and Medical Oncology, University Medical Center Göttingen, Göttingen, Germany

## Abstract

The karyotype represents one of the main cornerstones for the International Prognostic Scoring System (IPSS) and the revised IPSS-R (IPSS-R) that are most widely used for prognostication in patients with myelodysplastic syndromes (MDS). The most frequent cytogenetic abnormalities in MDS, i.e. del(5q), -7/del(7q), +8, complex karyotypes, or −Y have been extensively explored for their prognostic impact. The IPSS-R also considers some less frequent abnormalities such as del(11q), isochromosome 17, +19, or 3q abnormalities. However, more than 600 different cytogenetic categories had been identified in a previous MDS study. This review aims to focus interest on selected rare cytogenetic abnormalities in patients with MDS. Examples are numerical gains of the chromosomes 11 (indicating rapid progression), of chromosome 14 or 14q (prognostically intermediate to favorable), -X (in females, with an intermediate prognosis), or numerical abnormalities of chromosome 21. Structural abnormalities are also considered, e.g. del(13q) that is associated with bone marrow failure syndromes and favorable response to immunosuppressive therapy. These and other rare cytogenetic abnormalities should be integrated into existing prognostication systems such as the IPSS-R. However, due to the very low number of cases, this is clearly dependent on international collaboration. Hopefully, this article will help to inaugurate this process.

## Introduction

Myelodysplastic Syndromes (MDS) representing clonal hematopoietic stem cell disorders were shown to be highly heterogeneous from clinical, phenotypic, cytogenetic,^1^,^2^ and, more recently, from molecular genetic aspects.^3^–^5^ Cytogenetic abnormalities are detectable in 40–60% of patients with *de novo* MDS and in up to 90% of patients with therapy-associated MDS (t-MDS) or secondary MDS.^6^ Together with the bone marrow blast percentage and peripheral blood values (hemoglobin, neutrophils, thrombocytes), the karyotype of the hematopoietic cells provides the basis for the IPSS-R (international prognostic scoring system)^2^,^7^ and is essential for therapeutic decision making in patients with this heterogeneous disorder. This is of utmost importance considering the range of possible therapeutic strategies^8^ including purely supportive concepts, treatment with drugs including lenalidomide and demethylating agents (azacitidine and decitabine), or allogeneic hematopoietic stem cell transplantation (HSCT) for high-risk MDS patients.^9^ The karyotype furthermore has an important role establishing the diagnosis of MDS. During the course of the disease, karyotyping contributes to assess the response to therapy or may identify clonal evolution as a sign of progression.^10^

The most frequent cytogenetic abnormalities in MDS such as del(5q), -7/del(7q), +8, complex karyotypes, or −Y have been extensively explored for their prognostic impact. Some less frequent isolated cytogenetic abnormalities have been characterized for their impact on prognosis such as del(11q) (very good prognosis), isochromosome 17q or +19 (intermediate prognosis), or inv3/t(3q)/del(3q) (poor prognosis).^2^ These results found already entrance in the IPSS-R.^7^ However, there is a much larger variety of cytogenetic abnormalities in MDS. In a previous study^1^ investigating a large cohort of more than 2,000 patients with MDS, a total of 684 different cytogenetic categories were identified. The wide spectrum of less frequent cytogenetic categories includes e.g. gains of chromosomes 1 or 1q, 14 or 14q, gains or losses of chromosome 21, or loss of one X-chromosome.^1^ Many of those rare cytogenetic abnormalities are associated with distinct prognostic profiles. So far, these rare cytogenetic abnormalities only found limited attention in MDS studies. Aiming to focus interest on this issue, this review article discusses the role of several selected rare cytogenetic abnormalities (Table 1) in patients with MDS. Cytogenetic aberrations that had already been considered within the IPSS-R^2^,^7^ (i.e. cytogenetic abnormalities that were detected in more than 10 patients in the large cohort of patients with MDS were representing the basis for the cytogenetic scoring system of the IPSS-R)^2^ were not included in this review article. As a complete summary of all relevant rare abnormalities in MDS was beyond the scope of this article, the authors focused on a selection of abnormalities that were considered to be most interesting due to their prognostic relevance, due to specific biologic characteristics, or due to associations with specific molecular mutations.

In general, most frequent in MDS are cytogenetic losses resulting from monosomies or deletions, mostly involving chromosomes 5, 7, 20 or Y. The gain of genetic material with the occurrence of total or partial trisomies (e.g. of chromosomes 1, 8, 11, and 21) is less frequent. Unbalanced translocations may also cause losses or gains of genetic material. Different from AML balanced structural abnormalities occur only rarely in MDS. Some of the most important rare cytogenetic abnormalities in MDS are discussed in detail in the following, focusing on their frequency, their diagnostic meaning, their molecular background or associations and their impact on the prognosis according to the literature and own findings.

### Chromosomal Losses

#### Loss of the X-chromosome

Acquired non-constitutional loss of a sex chromosome (Y-loss in males, X-loss in females) is an age-related phenomenon, but can also occur in association with hematological malignancies.^11^–^14^ Usually, aberrations of the sex chromosomes are numerical losses or gains affecting the whole chromosome. Structural changes such as partial deletion of X are very rarely detected as part of unbalanced translocations or very complex aberrations. The loss of the Y-chromosome as a sole abnormality belongs to the more frequent cytogenetic abnormalities in MDS and is associated with a very good prognosis.^2^ In contrast, an acquired loss of the X-chromosome in females is a rare abnormality in MDS and AML and is described to occur as a single abnormality in 0.2 – 0.3% of patients with these myeloid malignancies only^2^,^15^,^16^ and in up to 1.5% in combination with other abnormalities.^17^ As a sole aberration, loss of X-chromosome in MDS is associated with an intermediate prognosis, a median overall survival (OS) of 16 months and a median leukemia-free survival of 14 months.^2^ As a constitutional abnormality, a monosomy X defines Turner’s syndrome,^18^,^19^ but the respective syndrome does not seem to be associated with an increased risk of hematological malignancies.^20^–^22^

By FISH a suitable probe for the detection of the sex chromosomes is easily available and can be performed on different samples (bone marrow, CD34+ myeloid peripheral blood cells, CD3+ peripheral T-cells).^13^,^23^,^24^ Further cytogenetic analyses of e.g. PHA-stimulated lymphocytes are needed^11^ to distinguish a constitutional monosomy X from an acquired loss of the X-chromosome restricted to the hematopoietic cells.

#### 13q Deletion

**Figure 1:**
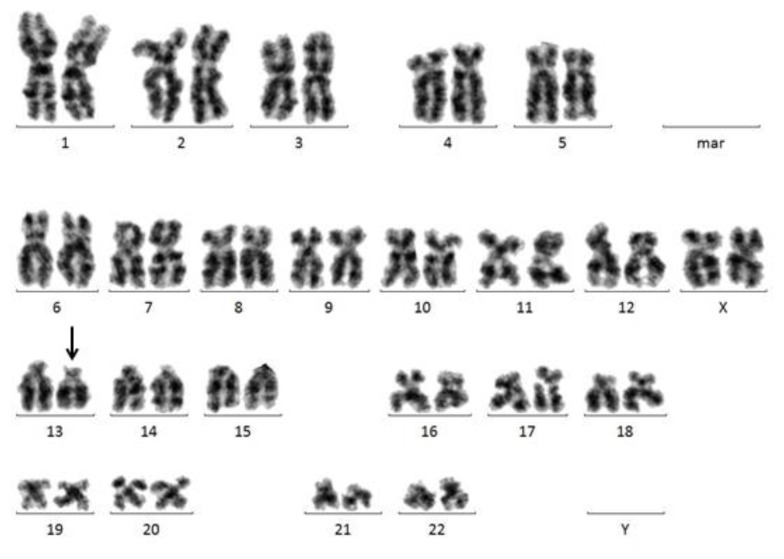
Isolated 13q deletion in a female patient with MDS.

Deletion of 13q occurs in different hematological malignancies, i.e. in lymphatic malignancies such as chronic lymphocytic leukemia (CLL) or multiple myeloma, but may also be observed in myeloid malignancies such as chronic myeloid leukemia (CML), myeloproliferative neoplasms (MPNs), or MDS (Figure 1). The *RB1* gene that had been first identified in patients with retinoblastoma^25^ was shown to be located within the common deleted region in leukemic cells carrying a del(13q).^26^ The *RB1* gene is a tumor suppressor gene that is involved in cell-cycle control and in the process of cell differentiation.^27^ FISH with probes for *RB1* is a useful adjunct to cytogenetics when abnormalities of 13q14 are detected.^28^ In MDS, the frequency of 13q deletion was given around 2%.^27^ In patients with myeloid malignancies and occurrence of 13q deletion, a strong association to therapy-associated MDS or AML has been described.^27^

It has been suggested that unclassified MDS (MDS-U) with del(13q) was a benign bone marrow failure subset characterized by good response to immunosuppressive therapy and a high prevalence of PNH clones.^29^–^31^ Hosokawa *et al.* investigated 22 patients showing bone marrow failure with a sole del(13q) or with a del(13q) and additional cytogenetic abnormalities. All del(13q) patients were diagnosed with MDS-U. PNH clones were detected in 19 patients. Strikingly, all 14 patients with del(13q) alone and 2 of 5 patients with additional cytogenetic abnormalities responded well to immunosuppressive therapy with favorable 10-year OS rates of 83% and 67%. Only two patients with additional cytogenetic abnormalities developed s-AML.^29^ To further investigate this phenomenon, Holbro *et al.* analyzed 86 patients with aplastic anemia (AA) with available cytogenetic results. Six patients (7%) showed a del(13q). All but one of these patients showed evidence of PNH clones. Also in this series, there was a favorable response to immunosuppressive therapy and all patients but one were still alive after a median follow-up of 105 months. None of the patients with del(13q) developed leukemic transformation. Thus, the patients with bone marrow failure and del(13q) from both cohorts (Holbro *et al.*; Hosokawa *et al.*) showed similar characteristics and outcomes. These reports suggest that MDS-U with del(13q) may be seen as an overlap condition between MDS and AA.

#### Monosomy 21

Monosomy 21 (-21) as a sole abnormality is rare in primary MDS with a frequency of 0.3%.^2^ Monosomy 21 may also occur in combination with one more cytogenetic aberration, reaching a frequency of 0.5% in a large series of patients with primary and secondary MDS.^1^ The frequency of -21 as part of a complex karyotype is not well defined in the literature, possibly due to the small number of cases. The knowledge regarding the prognostic impact of a sole -21 is limited. In a series of 8 patients with a -21 as a sole abnormality, the median OS was calculated as 32 months and the leukemia-free survival as 31.3 months,^2^ which was in good accordance with the previous study of Haase *et al.*.^1^ Thus, an isolated -21 would fit into the intermediate risk group according to the IPSS-R.^7^ However, due to the very low number of patients, this prognostic assessment needs to be considered with caution. Monosomy 21 is associated with low peripheral blood values and elevated bone marrow blasts. In 8 patients with an isolated -21, reported by Schanz *et al.*, the median hemoglobin level was 7.2 g/dl, the median platelet count was 35/nl and the median ANC count 2.2/nl, respectively. The median bone marrow blast count was 13%.^2^ The finding of a -21 by karyotyping, sometimes, does not reflect a real monosomy but a technical artifact that mimics the respective abnormality. Loss of chromosome 21 may occur randomly by preparing the cells for chromosome banding analysis. Thus, the finding of a monosomy 21 by chromosome banding analysis should be confirmed by other techniques like FISH at least if it appears in a small mosaic.^32^

### Chromosomal Gains

#### Trisomy 11

Isolated trisomy 11 (+11) is a very rare abnormality in MDS. The frequency was described between 0.2 and 0.3% of all MDS cases.^2^,^33^–^35^ Wang *et al*.^33^ report on a retrospective study with a duration of 15 years including 17 out of 5,000 MDS patients with +11 as a sole abnormality (n=10) or as part of a non-complex karyotype (n=7). The additional abnormalities contained trisomies of chromosomes 2, 8, 10, 19 or 22 without any typical poor-risk abnormalities such as chromosome 7 abnormalities. MDS patients with trisomy 11 showed a median survival time of 14 months only and a high frequency of disease progression. Within a median interval of 5 months 69% of the affected patients developed s-AML, the remaining patients progressed to advanced MDS stages.^33^

Trisomy 11 can also occur in patients with AML. MDS and AML patients with +11 were reported to show early stem cell features with a low degree of maturation.^33^,^35^,^36^ AML patients with trisomy 11 as isolated abnormality or within a non-complex aberrant karyotype were reported to show as well adverse outcomes with a median OS of only 9 months.^33^ Alseraye *et al*.^35^ published a retrospective study including more than 20,000 AML patients in a 10-years-period and found 18 patients (0.09%) with isolated +11: 14 patients with *de novo* AML and 4 patients with s-AML following MDS who showed a median OS of 5 months and an aggressive course of the disease. Obviously, +11 does not show any specific association to a certain WHO (WHO) or FAB MDS subtype or secondary or therapy-related MDS.^33^

Trisomy 11 affects the *MLL* gene on 11q23.^37^ Sixty-four percent of AML and 50% of MDS patients with +11 showed *MLL* partial tandem duplications (*MLL*-PTDs).^33^ Twenty to twenty-five percent of AML with +11, but no MDS patients with this cytogenetic abnormality presented an additional *FLT3*-ITD mutation.^33^,^38^

Because of the very low frequency of isolated +11 in MDS, it was so far assigned to the intermediate risk category according to the IPSS and the IPSS-R, respectively.^2^,^7^,^39^ According to the published literature, trisomy 11 seems to be associated with an aggressive course of the disease, short OS, and rapid progression or leukemic transformation.

#### Trisomy 13

Trisomy 13 (+13), especially as solitary abnormality, is very rare, however it has been observed recurrently in myeloid neoplasia, mostly in AML, primary myelofibrosis, atypical CML and MDS.^40^ Isolated +13 in a patient with MDS RAEB was first reported in 1989.^41^ A second case report – also MDS RAEB - followed in 1990.^42^ In the same year +13 was identified as a rare but recurrent abnormality in *de novo* acute leukemia being observed in 8/621 (1.3%) consecutive patients (5 AML, 1 acute mixed lineage leukemia, 1 ALL, 1 acute undifferentiated leukemia). The survival of these patients was poor with a median of 9.5 months (range: 0.5 to 14.7 months).^43^ The United Kingdom Cancer Cytogenetics Group published a survey report on patients with +13 and myeloid malignancy. Of the 28 patients reported 5 (18%) had MDS (1 RA, 1 RAEB, 5 RAEB-t) while the others had AML. In general the OS of the entire group was poor (median: 3.0 months, range: 1–96), however no prognostic data were available for single patients with MDS.^44^ In a multicenter cytogenetic study our group observed isolated +13 in 4 (0.2%), with one additional abnormality in 4 (0.2%) and in 8 (0.4%) patients as a component of complex changes in a cohort of 2,072 patients with MDS.^1^ This is contrasting the statement in the “Huret Atlas” saying that in the majority of cases +13 is the only cytogenetic abnormality.^40^ In AML, trisomy 13 was shown to be closely associated with *FLT3* overexpression and cooperating *RUNX1* mutations.^45^,^46^

In a large series published by the Mayo Clinic in Rochester +13 was detected in 0.2% (n=9) of all MDS and 0.7% (n=15) of all AML patients. Furthermore one patient with primary myelofibrosis, one with CMML and one with pernicious anemia had +13, too. There was a clear gender imbalance with 21/27 males. The median age was 73 years. In 96% of these cases, +13 was isolated. In the 9 MDS cases, the majority showed blast increase and disgranulopoiesis with hypogranulation. Furthermore, 6/9 patients showed small monolobated megakaryocytes remembering the situation in del(5q) MDS. Two patients with higher risk MDS were treated with hypomethylating agents but did not respond. Blood values (medians) for the whole +13 cohort were Hb = 9.0 g/dl (6.2–13), leukocytes = 5.3/nl (1.0–264.8) and platelets = 87/nl (11–312). The whole trisomy 13 cohort (summarizing the MDS and AML patients) revealed a median survival of 5.8 months (range: 0.5 – 24).^47^ In 2 older male patients with AML and isolated +13 treatment with high dose lenalidomide resulted in a sustained morphologic and cytogenetic complete remission in this otherwise poor risk cytogenetic subset of AML not responding to standard high-dose chemotherapy.^48^

In conclusion, +13 is a very rare condition in MDS, occurring in 0.2 to 0.8% of patients, mostly as an isolated change. In AML +13 is a bit more frequent with an incidence between 1 and 2%. The majority of patients have isolated +13. Most patients are over 70 years old and are males. Typically, the MDS is advanced with blast excess and moderate pancytopenia. Although most survival data relate to AML cases, the prognosis is bad with median survival ranging between less than six months and one year. Patients with AML and +13 do not respond to standard intensive chemotherapy, and hypomethylating agents for MDS patients also might be inefficient although database is not satisfying concerning this issue. However, high-dose lenalidomide could be an option. On a molecular level, +13 is associated with an overexpression of *FLT3* located at the triplicated chromosome. Furthermore, there is a close association between +13 and mutations of *RUNX1*.

#### Trisomy 14/14q

Trisomy 14, in general, is extremely rare in hematologic malignancies but shows a clear association with myeloid neoplasia.^49^ Trisomy 14/14q has been mainly observed in MDS and CMML; more rarely it has been seen in AML and in atypical MDS/MPN overlap syndromes.^49^–^52^ Most reports in the literature refer to single cases or smaller series of patients. In the largest series (n=16) published so far, 10 patients presented with isolated complete trisomy 14, in one case trisomy 14 represented the second evolutionary step in a patient with loss of the Y-chromosome as primary change. Finally, 5/16 patients showed an isolated isochromosome 14q – i(14)(q10) – for the long arm, resulting in trisomy 14q. In this series, 7/16 patients had MDS (including 4 RAEB-1/-2, 4 CMML, 4 AML and one atypical MDS/MPN overlap syndrome).^50^ The median age was ranging between 67 and 72 years. There was a male preponderance with roughly 70% of patients being of the male gender.

In a multicenter cytogenetic study we observed +14 in 3 (0.1%) patients, with one additional abnormality in 4 (0.2%) and in 9 (0.4%) patients with complex changes out of 2,072 patients of the entire cohort.^1^ To the best of our knowledge, this is the only published report^1^ on the frequency of trisomy 14 as yet. It has to be mentioned that cases with i(14)(q10) were not included in our dataset. The cumulative incidence combining trisomy 14 and i(14)(q10) may be one-third higher.

Hematologic findings in patients with trisomy 14/14q are inconsistent. Mancini reported a tendency to normal or increased platelets while in the case collection of Cui patients with MDS showed a broad range of platelet levels (23 – 465/μl, median 79/μl). Remarkably thrombocytosis was only observed in 2 RARS cases, whereas in the remaining five MDS cases (4 RAEB-1/-2 and 1 RCMD) platelets were low to normal (23 – 156/μl, median 75/μl). Patients with CMML showed normal to increased platelet counts (160 – 626/μl, median 198/μl). Hb values ranged between 4.0 g/dl and 12.0 g/dl (median 9.8 g/dl) in the review of Mancini with comparable data (median 10.0 g/dl) in the series of Cui.^50^,^51^ Leukocytes ranged between 2.6/nl and 9.2/nl (median 3.8/nl) in Mancini’s series and between 1.6 and 8.8/nl (median 4.2/nl) in Cui’s cohort thus being moderately reduced to normal.

In a literature review, survival data from 18 patients suggest an intermediate prognosis.^51^ These data are supported by the findings of Cui *et al.* who reported a median survival of 28 months in MDS, 31.5 months in MDS/MPN and nine months in AML.^50^

The only systematic molecular investigation (*KIT*, *RAS*, *FLT3*, *NPM1*, *JAK2*) in this cytogenetic subgroup identified *FLT3-ITD* in 1/6 MDS at diagnosis later transforming to s-AML and *KRAS*-mutation in 1/7 MDS patients at the time of leukemic transformation. Thus, the molecular background in these cases remains to be clarified.^50^ More comprehensive analyzes are needed, however surely hampered by the scarcity of trisomy14/14q.

Taken together trisomy 14/14q occurs in less than 0.5% of patients with MDS as isolated change. The abnormality seems to be an early event, and the affected cell clones seem to be genetically stable with a low tendency to acquire additional changes and a low tendency to leukemic transformation.^50^ The abnormalities typically can be seen in older male MDS patients with and without blast excess. The prognosis seems to be intermediate to good. Blood counts typically show normal leukocytes or mild leukocytopenia, moderate anemia and moderately reduced to normal platelets with the exception of thrombocytosis in RARS. In CMML, platelets are not decreased. The molecular background is unclear. Especially the role of oncogenes located at 14q needs further elucidation.

#### Trisomy 21

**Figure 2:**
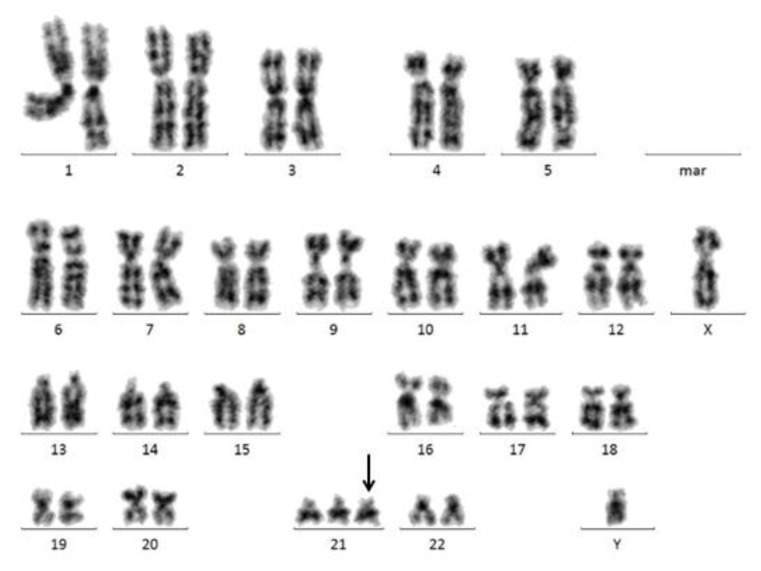
Isolated trisomy 21 in a male patient with MDS.

Trisomy 21 (+21) is well known in the context of Down’s syndrome, associated with a marked risk to develop AML during childhood. However, besides this hereditary disease, +21 may also occur as a clonal somatic abnormality in several hematologic disorders. In adult *de novo* AML, trisomy 21 occurs in around 3% of patients.^53^ In MDS, +21 as a single abnormality (Figure 2) is occurring more rarely. In a series of 968 patients published by Solé *et al.*, isolated +21 was detected in 0.8% of patients and showed a significant association with CMML, where 3.5% of patients showed +21 as sole abnormality.^54^ Further publications found a comparable incidence, calculated as 1.1% of patients showing +21 within a non-complex karyotype. Based on a cohort of 2,901 patients, the incidence of +21 as an isolated abnormality was 0.3%, assigning this abnormality to the group of rare abnormalities in MDS. Based on nine patients, the authors described an association with a low ANC (median 1.9/nl) and a slightly decreased platelet (median 105/nl) and hemoglobin (9.1 g/dl) level. The median blast count in these patients was 6%, indicating an association with higher risk MDS.^2^ The median OS was 100.8 months in +21 within a non-complex karyotype.^1^ Other publications stated a median OS of 13.9 months^54^ and 21.5 months 2 respectively, for patients showing isolated +21. The median time to AML evolution was 100.7 months in the publication of Schanz *et al.*^2^ Solé *et al.* stated a cumulative AML risk of 25% after one year and 50% after five years.^54^ Taken these results together, the prognostic impact of an acquired, isolated +21 in patients with MDS remains unclear und has to be stated as intermediate until a higher number of patients were analyzed.

The molecular background of patients showing +21 in myeloid malignancies remains undefined as yet. *RUNX1* (=*AML1*), located on chromosome 8q22, commonly involved in t(8;21)/*RUNX1-RUNXT1* in AML, was shown to be also point mutated in patients with myeloid malignancies like AML, MDS and MPN with +21.^55^ A Japanese group found a poor prognostic impact of intragenic *RUNX1* mutations in MDS but did not describe a correlation with +21.^56^

### Structural Abnormalities

#### Gain of chromosome 1q

Gain of 1q resulting in complete or partial trisomy 1q can be due to duplications, to the formation of an isochromosome i(1q), to the gain of a deleted or a whole chromosome, or to unbalanced translocations with different partner chromosomes as demonstrated in an MDS case series presented by Fonatsch *et al*.^57^

**Isochromosome 1q** [i(1q)] may occur in different hematological malignancies such as ALL or lymphoproliferative disorders, or in solid tumors.^58^ In myeloid disorders, i(1q) shows very rare occurrence.^58^ Pawarode *et al.* reported on a male patient developing t-MDS with isolated i(1q) 14 years after therapy of acute promyelocytic leukemia (APL). There was no blast increase, and the t-MDS showed only slow progression under supportive therapy.^58^ Occasional cases with i(1q) as isolated abnormality have also been reported as *de novo* MDS, e.g. a 20-year-old female patient with a hypocellular bone marrow who received allogeneic HSCT.^59^ Fonatsch *et al.* reported on a 24 years old male patient with RAEB-T and isolated i(1q) who remained clinically stable over ten months.^57^ Prognostic assessment of i(1q) is difficult due to the rare occurrence.

**Duplication of 1q** mainly occurs as a secondary event in MDS. Occasionally, dup(1)(q21q32) has been documented as a sole cytogenetic abnormality in MDS patients. Alfaro *et al.* reported on two MDS patients with a sole dup(1)(q21q32). One patient showed a clonal cytogenetic evolution with an additional +8; the other patient evolved to s-AML.^60^ The authors considered duplication of 21q to be prognostically adverse in MDS.

Furthermore, various **unbalanced translocations** may lead to a gain of 1q. Derivative translocation der(1;5) was described mostly in patients with AML, but also in patients with CML, MPNs, or MDS. The breakpoints of der(1;5) vary from 1q11 to 1q43, with a clustering to 1q21-23, and the 5q breaks occurred in 5q11 to 5q35, with preponderance in the distal 5q3 region. Derivative translocation der(1;5) was either reported as isolated abnormality or in combination with other cytogenetic abnormalities.^61^ According to a collection of two own cases and a review of the literature, Johansson *et al.* suggested that the prognostic impact of the respective abnormality was poor as most patients included in this report had died.^61^ Lunghi *et al.* summarized several MDS cases with der(1)(t;16) from the literature. Male preponderance was evident. Patients showed different MDS subtypes with or without blast increase, but a high transformation rate to s-AML was noted in 4 out of 7 patients. As shown by FISH analyzes, the respective translocation was resulting in a gain of 1q and loss of 16q.^62^ Another rare unbalanced translocation in MDS resulting in a gain of 1q is der(1)(1;13)(q21;p12).^57^

Hypothetically, the major consequence of these unbalanced translocations is the chromosomal imbalance resulting from the gain of the long arm of chromosome 1q and the loss of genetic material from the partner chromosome rather than the chromosomal break *per se*.^62^ This argument is further underlined by the fact that the breakpoints within 1q show considerable variation depending on the translocation partners. Based on the analysis of a large cohort of 968 patients with MDS, Sole *et al.* came to the conclusion that MDS with 1q involvement shows a poor outcome when compared with the overall series of patients. However, due to the rare occurrence of this abnormality the authors recommended caution with regards to this interpretation.^54^

**Figure 3:**
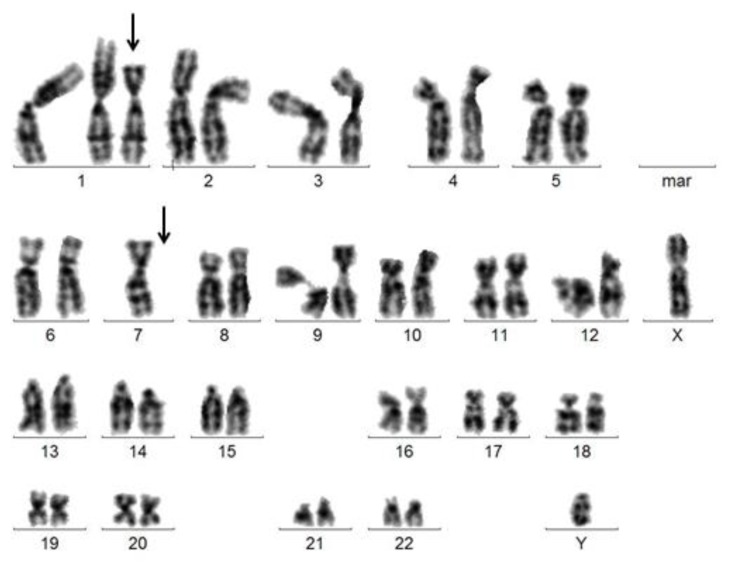
Karyogramme of a male patient with MDS RCMD showing monosomy 7 and an additional derivative chromosome der(7)t(1;7)(q10;p10): 46,XY,+der(7)t(1;7)(q10;p10),-7. The additional chromosome der(7)t(1;7)(q10;p10) and the missing chromosome 7 are marked by arrows.

The most frequently reported unbalanced translocation in this context is the derivative translocation **der(7)t(1;7)(q10;p10)** that represents an unbalanced whole-arm translocation between the centromere-near regions of 7p and 1q and results in unbalanced gain of 1q and loss of 7q (Figure 3). It mostly occurs in MDS, but may also occur in AML (often of secondary origin) or myeloproliferative neoplasms (MPNs) such as myelofibrosis. The der(7)t(1;7)(q10;p10) has been described to occur in 1–3% of MDS cases, more frequently in males.^63^,^64^ In half of all cases, der(7)t(1;7)(q10;p10) occurs as sole abnormality, in a third of cases it is accompanied by numerical gains such as +8 or +21; additional structural abnormalities were described in around 15% of affected cases.^63^ Even in case of multiple cytogenetic subclones, it could be shown that der(1;7) affects all abnormal metaphases. Furthermore, the der(1;7) can be identified in all affected patients already at first diagnosis of the disease^65^ which suggests a high level of genetic stability for the respective cytogenetic abnormality.

A poor prognostic impact and a high risk of transformation to s-AML has been described in MDS patients carrying der(1;7). Slovak *et al.* compared the prognosis of der(1;7) positive MDS patients with patients with chromosome 7 abnormalities (-7 and 7q-; both are known to be prognostically highly adverse) and found no significant differences with regard to leukemic transformation rate or 5-year survival between these different cytogenetic risk groups.^64^ On the other hand, Sanada *et al.* retrospectively compared a cohort of 77 patients suffering from different myeloid malignancies (MDS, AML, MPNs) all carrying der(1;7) with patients with -7 or 7q. The MDS patients with a der(1;7) showed significantly lower blast counts, higher hemoglobin levels, and slower progression to s-AML as compared with the cases carrying other chromosome 7 abnormalities.^65^ However, as the median survival of the MDS patients with a der(1;7) was only 23.7 months in this study^65^ there was no doubt that the respective abnormality confers an adverse prognosis in patients with MDS. Our unpublished data confirm the significant better outcome of MDS patients with a der(1;7) as compared with patients with -7 or 7q-.^66^ Hsiao *et al.* documented frequent occurrence of previous chemo- or radiotherapy or additional cytogenetic changes in patients with MDS or AML with der(1;7).^67^ Westman *et al.* described an increased frequency of isocitrate dehydrogenase (*IDH1* and *IDH2*) mutations in t-MDS/AML patients with der(1;7) and other +1q abnormalities.^68^

#### 11q23/*MLL* translocations

Reciprocal translocations of 11q23, involving the *MLL* gene, are frequent in adult and pediatric acute lymphoblastic and myeloid leukemia. Rearrangements of the *MLL* gene with several partner genes result in various effects on hematopoietic stem cells, especially differentiation anomalies. Actually, more than 70 translocation partners have been identified.^69^ On the molecular level, the *MLL* gene may also be affected by partial tandem duplications (*MLL*-PTDs). 11q23/*MLL* translocations are seen in around 22% of patients with ALL which is much more frequent as compared to AML with only 5% of patients being affected.^70^ In patients with ALL, t(4;11) is most frequent, followed by t(11;19). In AML, t(9;11) and t(10;11) are most commonly detected.^70^ In therapy-related AML/MDS, 11q23/*MLL* translocations are strongly associated with a previous exposition to topoisomerase-II inhibitors (e.g. etoposide).^71^ About 5% of all patients showing 11q23/*MLL* translocations suffer from myelodysplastic syndromes.^72^ 11q23/*MLL* translocations are very rare in primary MDS and can be found in 0.5% of patients as part of a non-complex karyotype.^1^ The frequency of 11q23/*MLL* translocations as sole aberrations in MDS is even lower with 0.2% of all patients.^2^ 11q23/*MLL* translocations are correlated with the WHO subtypes RA or RAEB-1.^72^ In the 7 patients with sole 11q23/*MLL* translocations described by Schanz *et al.*, these abnormalities were associated with a low hemoglobin level (7.9 g/dl) but a normal platelet (140/nl) and ANC (6.3/nl) count. The median bone marrow blast count in these seven patients was 4.0%. A higher frequency of 11q23/*MLL* translocations was described by Solé *et al.* in a study based on 968 patients with MDS. In this cohort, sole 11q23/*MLL* translocations were described in 6% of patients.^54^ The authors found no correlation with FAB subtypes.

Regarding the prognostic impact of 11q23/*MLL* translocations in MDS, Solé *et al.* found a median OS of 26 months and a cumulative risk for developing acute leukemia of 40% to 1 year and 92% to 5 years,^54^ but did not discriminate between the different types of 11q23/*MLL* translocations. In another study, the median OS of patients with 11q23/*MLL* translocations occurring within a non-complex karyotypes was 20 months.^1^ In this study, primary as well as therapy-associated MDS patients were included. The largest series of patients with a primary, untreated MDS described a median OS of 26.7 months and a median AML-free survival of 78 months.^2^ However, also due to the low number of patients showing 11q23/*MLL* translocations in MDS, their real prognostic impact is not well defined for this hematologic entity. Consequently, as other rare single abnormalities, 11q23/*MLL* translocations were assigned to the intermediate risk group in the IPSS and also in the IPSS-R.^7^,^39^

## Conclusion

Due to the missing statistical validity caused by the low number of cases, rare cytogenetic abnormalities that are not considered specifically by the IPSS-R are assigned to the prognostically intermediate cytogenetic subgroup^2^ corresponding to 2.0 scoring points within this risk stratification system.^7^ Although the cytogenetic scoring categories of the IPSS-R include already more than 90% of patients with MDS,^2^ several patients remain for whom a correct cytogenetic classification is not available so far. Although a complete overview of all relevant rare cytogenetic abnormalities in MDS^1^ is beyond the scope of this review, this article provides examples for cytogenetic abnormalities that could be integrated into risk stratification systems. According to the above-cited studies and own experience, loss of the X-chromosome,^2^ loss^1^,^2^ and gain^2^,^54^ of chromosome 21, and 11q23/*MLL* translocations are associated with an intermediate prognosis. Trisomy 14 is prognostically intermediate to favorable.^50^,^51^ 13q deletion is associated with favorable response to immunosuppressive therapy.^29^,^30^ The prognostic impact of a der(7)t(1;7)(q10;or p10) is less adverse as compared to monosomy 7 or 7q deletion.^66^ Trisomy of chromosomes 11^33^ and 13^47^ mediates an unfavorable impact. These and other examples of prognostically relevant rare cytogenetic abnormalities emphasize the necessity to expand existing prognostic models and to optimize the IPSS-R.^2^,^7^

Finally, our working group would like to emphasize our interest in collecting additional cases with rare cytogenetic abnormalities with the aim to expand our database, to increase insights in the cytogenetic complexity of myelodysplastic syndromes, and to promote the exchange between hematologists interested in this issue.

## Figures and Tables

**Table 1:** Frequency of selected rare cytogenetic abnormalities (occurring as non-complex alterations either as isolated abnormalities or in combination with one additional abnormality) in a previous study investigating a total of 1,084 patients with *de novo* MDS (being part of the total study cohort consisting of 2,124 MDS patients).^1^

	Frequency related to all cases		
Cytogenetic abnormality	as non-complex abnormality	as isolated abnormality	Plus 1 add. abnormality
**Loss of chrom. X**	0.74%	0.37%	0.37%
**13q deletion**	0.74%	0.37%	0.37%
**Monosomy 21**	0.65%	0.28%	0.37%
**Trisomy 11**	0.92%	0.55%	0.37%
**Trisomy 13**	0.74%	0.37%	0.37%
**Trisomy 14/14q**	0.65%	0.28%	0.37%
**Trisomy 21**	2.12%	0.46%	1.66%
**der(7)t(1;7)(q10;p10)**	0.41%*	0.30%^2^	0.11%*
**11q23/*MLL* translocations**^2^	0.41%*	0.20%^2^	0.21%*

Chrom.: chromosome; add.: additional;

* unpublished data
